# Supplementary material for: End Sequence Analysis Toolkit (ESAT) expands the extractable information from single-cell RNA-seq data
Source: Genome Res. 2016 Oct;26(10):1397–410. doi: 10.1101/gr.207902.116 (PMC5052061; doi:10.1101/gr.207902.116)
Supplement: Supplemental Material [file supp_gr.207902.116_Supp_Materials.tar.gz › ESAT-esat_v0.1/doc/html/interfacebroad_1_1core_1_1multiplealignment_1_1_multiple_alignment_i_o-members.html]

ESAT: Member List


ESAT


- Main Page
- Related Pages
- Packages
- Classes
- Files

- Class List
- Class Index
- Class Hierarchy
- Class Members

All Classes Namespaces Files Functions Variables Enumerator Pages

- broad
- core
- multiplealignment
- MultipleAlignmentIO

broad.core.multiplealignment.MultipleAlignmentIO Member List

This is the complete list of members for broad.core.multiplealignment.MultipleAlignmentIO, including all inherited members.

|  |  |  |
| --- | --- | --- |
| getPreferredFileExtension() | broad.core.multiplealignment.MultipleAlignmentIO |  |
| load(String fileName) | broad.core.multiplealignment.MultipleAlignmentIO |  |
| load(InputStream in) | broad.core.multiplealignment.MultipleAlignmentIO |  |
| load(String fileName, List< String > sequencesToLoad) | broad.core.multiplealignment.MultipleAlignmentIO |  |
| load(InputStream in, List< String > sequencesToLoad) | broad.core.multiplealignment.MultipleAlignmentIO |  |
| write(BufferedWriter bw, MultipleAlignment ma) | broad.core.multiplealignment.MultipleAlignmentIO |  |
| write(BufferedWriter bw, MultipleAlignment ma, List< String > orderOfSequences) | broad.core.multiplealignment.MultipleAlignmentIO |  |


---

Generated on Fri Aug 15 2014 15:10:55 for ESAT by  

 1.8.7
